# Aliphatic Polyester Nanofibers Functionalized with Cyclodextrins and Cyclodextrin-Guest Inclusion Complexes

**DOI:** 10.3390/polym10040428

**Published:** 2018-04-11

**Authors:** Ganesh Narayanan, Jialong Shen, Ramiz Boy, Bhupender S. Gupta, Alan E. Tonelli

**Affiliations:** 1 Fiber and Polymer Science Program, North Carolina State University, Raleigh, NC 27695, USA; jshen3@ncsu.edu (J.S.); bgupta@ncsu.edu (B.S.G.); 2 Department of Textile Engineering, Namık Kemal University, Corlu/Tekirdag 59860, Turkey; rboy@nku.edu.tr; 3 Department of Textile Engineering Chemistry and Science, North Carolina State University, Raleigh, NC 27695, USA

**Keywords:** poly(lactic acid), poly(ε-caprolactone), cyclodextrins, cyclodextrin-inclusion complexes, controlled drug delivery, rapid dissolution, pseudorotaxanes, mechanical properties

## Abstract

The fabrication of nanofibers by electrospinning has gained popularity in the past two decades; however, only in this decade, have polymeric nanofibers been functionalized using cyclodextrins (CDs) or their inclusion complexes (ICs). By combining electrospinning of polymers with free CDs, nanofibers can be fabricated that are capable of capturing small molecules, such as wound odors or environmental toxins in water and air. Likewise, combining polymers with cyclodextrin-inclusion complexes (CD-ICs), has shown promise in enhancing or controlling the delivery of small molecule guests, by minor tweaking in the technique utilized in fabricating these nanofibers, for example, by forming core–shell or multilayered structures and conventional electrospinning, for controlled and rapid delivery, respectively. In addition to small molecule delivery, the thermomechanical properties of the polymers can be significantly improved, as our group has shown recently, by adding non-stoichiometric inclusion complexes to the polymeric nanofibers. We recently reported and thoroughly characterized the fabrication of polypseudorotaxane (PpR) nanofibers without a polymeric carrier. These PpR nanofibers show unusual rheological and thermomechanical properties, even when the coverage of those polymer chains is relatively sparse (~3%). A key advantage of these PpR nanofibers is the presence of relatively stable hydroxyl groups on the outer surface of the nanofibers, which can subsequently be taken advantage of for bioconjugation, making them suitable for biomedical applications. Although the number of studies in this area is limited, initial results suggest significant potential for bone tissue engineering, and with additional bioconjugation in other areas of tissue engineering. In addition, the behaviors and uses of aliphatic polyester nanofibers functionalized with CDs and CD-ICs are briefly described and summarized. Based on these observations, we attempt to draw conclusions for each of these combinations, and the relationships that exist between their presence and the functional behaviors of their nanofibers.

## 1. Introduction

Electrospinning is a widely utilized technique to fabricate nanofibers with intricate architectures, making this process highly suitable for tissue engineering and environmental, energy, sensor, textile, food packaging, and agricultural applications [1,2,3]. Reasons for the widespread use of nanofibers include their large surface to volume ratio with high porosities, and very low weight, compared to bulk fibrous materials [4,5]. In addition to their nano-scale dimensions, electrospun nanofibers are of special interest in biomedical applications, as they imitate the native extracellular matrix of physiologic tissues [4,6]. Another advantage of nanofibers is their capability of delivering drugs that are otherwise impermeable to physiologic tissues [7]. Aliphatic polyesters belong to the biodegradable polymers that are currently widely used in various biomedical applications, including for treating orthopaedic, oral and maxillofacial, and neurological ailments [4,8]. Despite their potential in various medical applications, aliphatic polyesters lack surface epitopes, have poor biomechanical properties (low modulus or brittleness, for example), and longer degradation times (polycaprolactone, for example), thereby necessitating the incorporation of additional molecules to modulate these characteristics [4].

**Figure 1:**
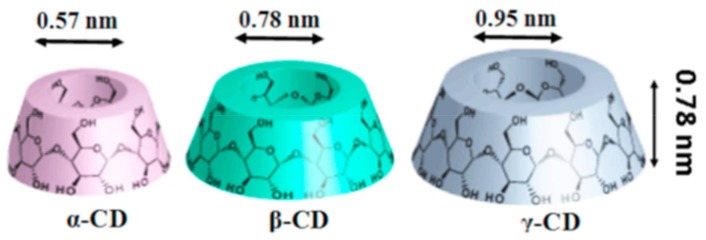
Schematic representations of the native cyclodextrins (CDs) (α-, β-, and γ-CDs). All three native CDs have a similar depth or height (7.9 Å); however, a key difference lies in their inner diameters, with α-, β- and γ-CDs having inner diameters of 5.7, 7.8, and 9.5 Å, respectively. Images adapted and reproduced from [11]. Copyright 2017 Elsevier Ltd.

Cyclodextrins (CDs) are cyclic oligosaccharides, essentially composed of α-D glucopyranose units joined by α-1,4 glycosidic bonds [9]. The most commonly used CDs are α-, β-, and γ-CDs. These have 6–8 glucopyranose units and all CD types have truncated cone-like structures with a hollow cavity [10]. Because of the presence of hydrophobic hollow cavities (Figure 1), CDs can encapsulate both small and large molecules, forming inclusion or, in the case of polymer guests, non-stoichiometric inclusion complexes, depending on the stoichiometry used between the host CD and guest molecules, resulting in the improved stability of the included guest molecules against heat, light, and other environmental conditions [11,12]. In addition, native CDs has been modified, resulting in modified CDs, such as random methyl, hydroxypropyl, peracetyl, sulfobutylether, and monochlorotriazinyl CDs, with improved water solubility compared to native CDs [11,13]. Both native as well as modified CDs have hydrophilic outer surfaces, in addition to hydrophobic cavities, facilitating secondary interactions with the included guest molecules [11]. 

Their unique structures make CDs capable of encapsulating smaller, as well as larger guest molecules (for example, polymers), resulting in the successful encapsulation of drugs, food ingredients (vitamins and fish oil, for example), light and air sensitive ingredients, as well as antibacterial/microbial agents [14,15]. Currently, a vast number of CDs are used in pharmaceutical formulations to improve the water solubility of hydrophobic drugs or to improve delivery rates to physiologic tissues, and/or in regenerative medicine [16,17,18,19,20,21,22,23]. Combining aliphatic polyesters with CDs, in particular, in nanofibrous form, can lead to interesting properties that can improve the versatility of these polymers for a variety of applications [24]. In this review, we summarize the various aliphatic polyester-based nanofibers, in particular those of biomedically relevant polymers, such as poly(ε-caprolactone) (PCL) and poly (lactic acid) (PLA), whose properties have been modified by either CDs or CD-inclusion complexes (CD-ICs), and that have been reported in the past decade. Although other biomedically relevant polymers, such as chitosan [25,26] and poly(butylene succinate-*co*-terephthalate) (PBST) [27], have also been modified with CDs or ICs. As those studies are far fewer, they are not addressed in this manuscript. 

## 2. Cyclodextrin Functionalized Aliphatic Polyester Nanofibers

### 2.1. Poly(ε-caprolactone) (PCL) Nanofibers Functionalized with Cyclodextrins or Small Molecule Inclusion Complexes

The CDs were simply combined with aliphatic polyester solutions (for example, poly(ε-caprolactone) (PCL) and poly(lactic acid) (PLA)), and subsequently electrospun, resulting in nanofibers with improved hydrophilicity and/or crystallinity profiles. Although this procedure has been employed for electrospinning various other polymer/CD combinations, such as polymethyl methacrylate [28,29,30], polystyrene [31,32,33], polyurethane [34,35], chitosan [36], poly(vinyl alcohol) [37,38,39], poly(ethylene terephthalate) [40], and polyethylene oxide [41], the key difference in aliphatic polyester/CD combinations is the lack of a suitable common solvent (for example, dimethyl formamide (DMF)), necessitating the use of binary solvent mixtures, such as chloroform or dichloromethane with DMF. However, the use of binary solvent mixtures is not without limitations, as such mixtures could lead to the inadvertent formation of ICs between polymers and CDs. It should also be noted that a common route for preparing ICs is by first dissolving CDs in water and guest molecules in organic solvents, and subsequently combining the two solutions. As binary solvent mixtures necessitate the use of organic solvents and water for dissolving guest and host molecules, respectively, mixing the solutions would inadvertently lead to the formation of ICs. 

**Figure 2:**
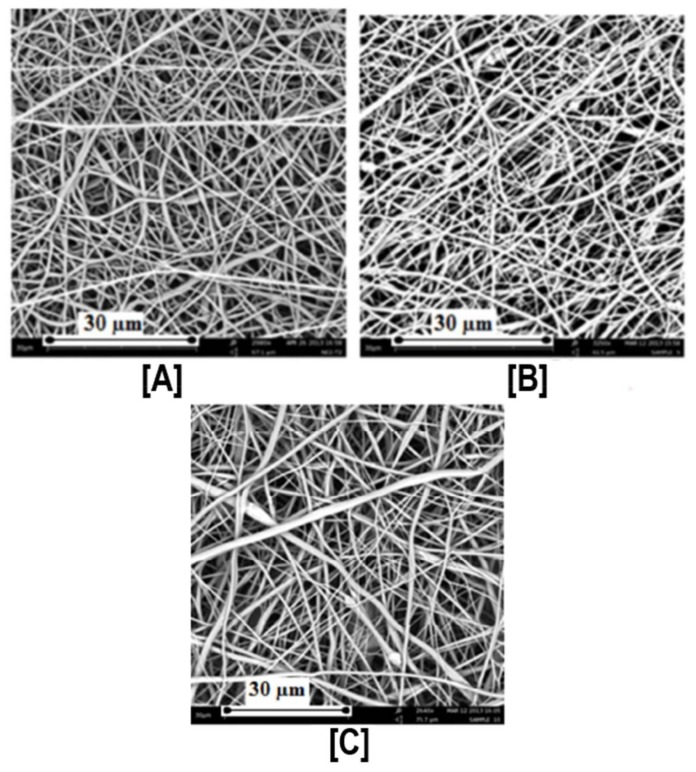
Scanning Electron Micrographs (SEM) of PCL and PCL/γ-CD functionalized nanofibers. By simply adjusting the PCL concentration from 14% to 12%, an increase in γ-CD loading (from 15% to upwards of 30%) was made possible without significant increases in the beaded structures in the nanofibers (**A**–**C**). Images adapted and reproduced with permission from [42]. Copyright American Chemical Society 2014.

**Figure 3:**
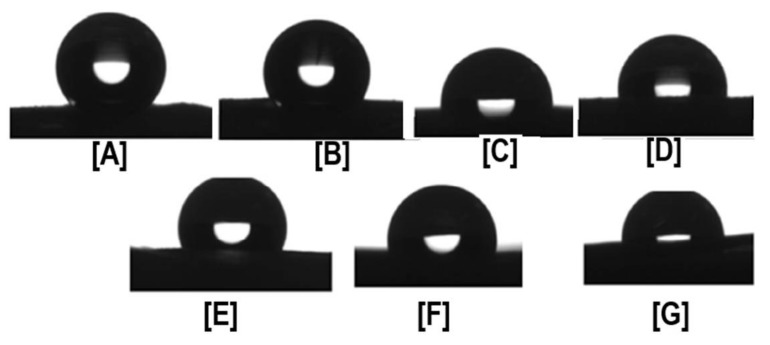
Water Contact Angle (WCA) values of PCL, PCL/α-CD, and PCL/γ-CD functionalized nanofibers. The neat PCL showed hydrophobic characteristics (WCA of 140°), while the WCA of functionalized nanofibers showed a marked decrease in hydrophobic characteristics, even with the addition of as little as 5% CD (WCA of PCL/5% α-CD and PCL/5% γ-CD are ~120°). With further addition, WCA plateaued at ~100°, depending on the CD used. Images adapted and reproduced with permission from [43]. Copyright American Chemical Society 2014.

**Figure 4:**
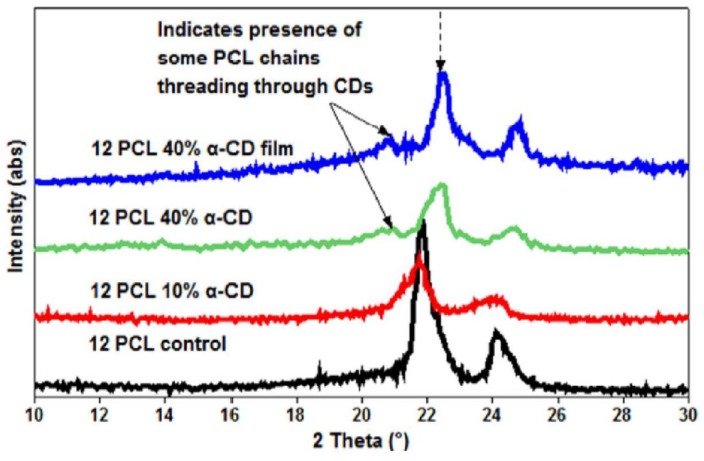
Wide-angle diffraction (WAXD) patterns of the electrospun PCL, α-CD functionalized electrospun PCL nanofibers, and PCL-α-CD film cast from solution. Neat PCL showed two peaks at 22° and 24° 2θ corresponding to (110) and (200) reflections. While the lack of any additional peaks in 10% α-CD containing PCL nanofibers, the presence of an additional peak at 20° 2θ and shifts in the PCL peaks, indicate the presence of some inclusion complex between PCL and α-CD. Images adapted and reproduced from [42]. Copyright American Chemical Society 2014.

Despite these challenges, our research group first reported the successful fabrication of PCL nanofibers containing α- and γ-CDs [42], and PCL with β-CDs [43]. In our first study, electrospinning of PCL with α- and γ-CDs was conducted, and subsequently the thermal, morphological, and water contact angle properties of the CD functionalized polymeric nanofibers were evaluated. With the incorporation of CDs, bead-free nano-sized fibers were obtained up to a loading of 15% (both α- and γ-CDs), with respect to PCL mass (Figure 2) [42]. Beyond the 15% addition, some thicker micro-sized fibers were obtained, although average fiber sizes remained at ~800 nm. With the addition of CD, major differences in the thermal properties of the PCL/CD composites, especially higher crystallinity and crystallization temperatures, were observed [42]. For instance, the crystallinity of PCL (from films), which is usually ~45%, increased dramatically to 53% and 65%, respectively, with the addition of 10% and 30% γ-CD. Furthermore, addition of CDs also resulted in a drastic reduction in the water contact angle (WCA) values of the composites. This effect of CD addition influencing WCA values was more prominent in PCL/α-CD composites at lower concentrations (5%, 10%, and 15%), and in PCL/γ-CD composites at higher concentrations (30% and 40%) [42] (Figure 3). Notwithstanding the thermal and morphological improvements, in addition to WCA values, thermogravimetric (TGA) and wide-angle diffraction (WAXD) analyses also showed the presence of some inclusion complexation between PCL and CDs. By virtue of lower weight loss at 370 °C, α-CD containing PCL composites illustrated the formation of IC between PCL and α-CD, compared to PCL/γ-CD at similar CD loadings [42]. In accordance with the TGA results, WAXD analyses of PCL/α-CD composites (10% CD loading) showed the presence of a diffraction peak at 2θ = 20°, in addition to PCL peaks at 2θ = 22° (110 reflection) and 24° (220 reflection), indicating the presence of PCL/α-CD in the complexed form, rather than in the neat state (Figure 4) [42]. In summary, this promising study reported the fabrication of PCL/α-CD composite nanofibers, with minimal formation of ICs between PCL and α-CD. 

**Figure 5:**
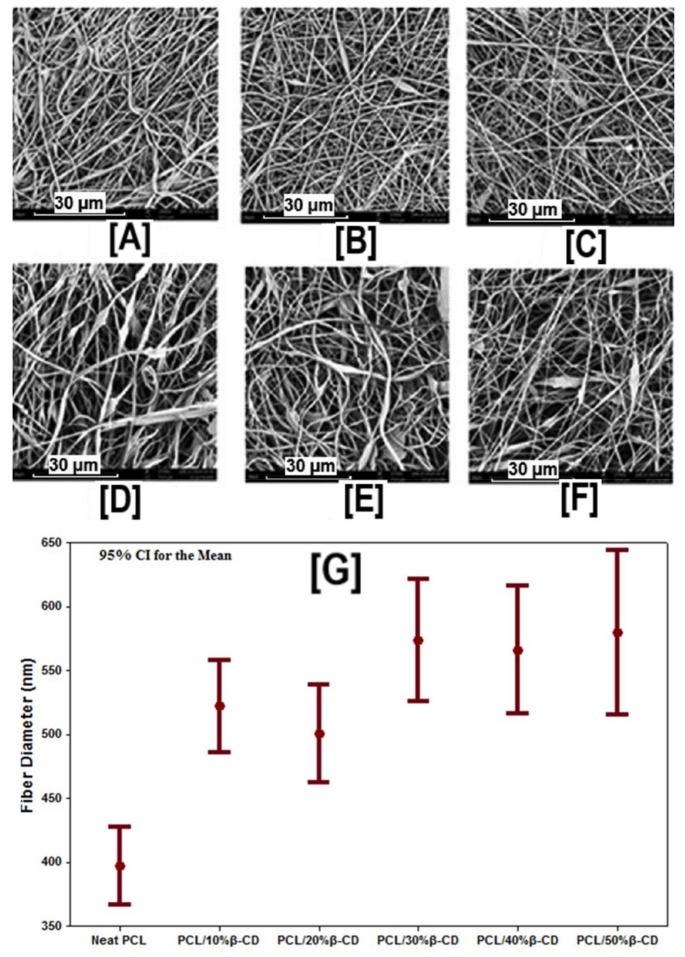
SEM images and average fiber diameters of β-CD functionalized PCL nanofibers at various β-CD loadings (0% to 50%). SEM images indicate the absence of beaded structures in PCL and PCL/β-CD nanofibers. While a marginal increase in fiber size was observed with the addition of β-CD (520 ± 200 nm for 10% β-CD vs. 400 ± 160 nm for neat PCL), further increases in β-CD concentration did not lead to further increases in fiber diameter. Images adapted and reproduced with permission from [43]. Copyright John Wiley and Sons 2015.

The formation of ICs between PCL and CDs (α- and γ-CD) can, to some degree, be expected, as both α- and γ-CD have shown an affinity towards IC formation [44,45,46,47,48,49]. However, due to the mismatch in the size between PCL and β-CD, such IC formation is not expected [50,51], leading to the formation of IC-free CD functionalized nanofibers. By employing the identical solutions and processing parameters used for fabricating PCL/α-CD and PCL/γ-CD nanofibers [42], PCL/β-CD nanofibers were fabricated via electrospinning [43]. Unlike PCL/α- or γ-CD nanofibers, CD loading of up to 50% was feasible without detrimental effects on fiber size (570 ± 310 nm) and morphology (bead-free nanofibers) (Figure 5). In addition, the absence of IC peaks from WAXD and higher mass loss in TGA analyses, showed a lack of IC formation between PCL and β-CD. More importantly, when incubated with wound odor emulating solutions containing butyric and propionic acids, X-ray photoelectron spectroscopy (XPS) and TGA analyses demonstrated higher volume capture of these molecules in PCL nanofibers containing β-CDs. This effect was observed, in particular, in those nanofibers containing higher loadings of β-CDs (>20%), compared to the control nanofibers (PCL), demonstrating the potential for these CD-containing nanofibers in wound dressings [43]. 

The encapsulation of small guest molecules in CD cavities enhances their stability, and in addition, facilitates melt or solution processing, irrespective of the nature of the guest molecules. Our research group has shown successful encapsulation of various guest molecules (triclosan, neomycin sulfate, antiblaze RD-1, nonoxynol-9, and azo-dyes) and subsequent melt processing with polymers [52,53,54,55,56]. By employing such a methodology, not only can the stability and controlled release of the guest molecules be ensured, but it also facilitates the fabrication of facile commercial products containing small guest molecules [56]. This approach is particularly advantageous when combined with nanofibers, as such products would, in addition, offer large surface areas with low weight, unlike those observed with bulk composite materials. 

The unique advantages of solid CD-ICs and nanofibers via electrospinning have been exploited in the past decade, resulting in materials suitable for a wide range of applications. For instance, α-tocopherol (α-TC), a potent form of vitamin-E is frequently used in wound dressings to promote wound healing. However, α-TC is not only poorly water soluble, but also susceptible to degrade under light and oxygen, for example [57]. When α-TC was encapsulated in β-CD, electrospun and carried by PCL nanofibers, it showed improvements in photostability (~6% higher than un-encapsulated form) and antioxidant activity, despite only a marginal increase in fiber diameter (345 ± 140 vs. 205 ± 115 nm for PCL/α-TC nanofibers without β-CD) [57]. 

**Figure 6:**
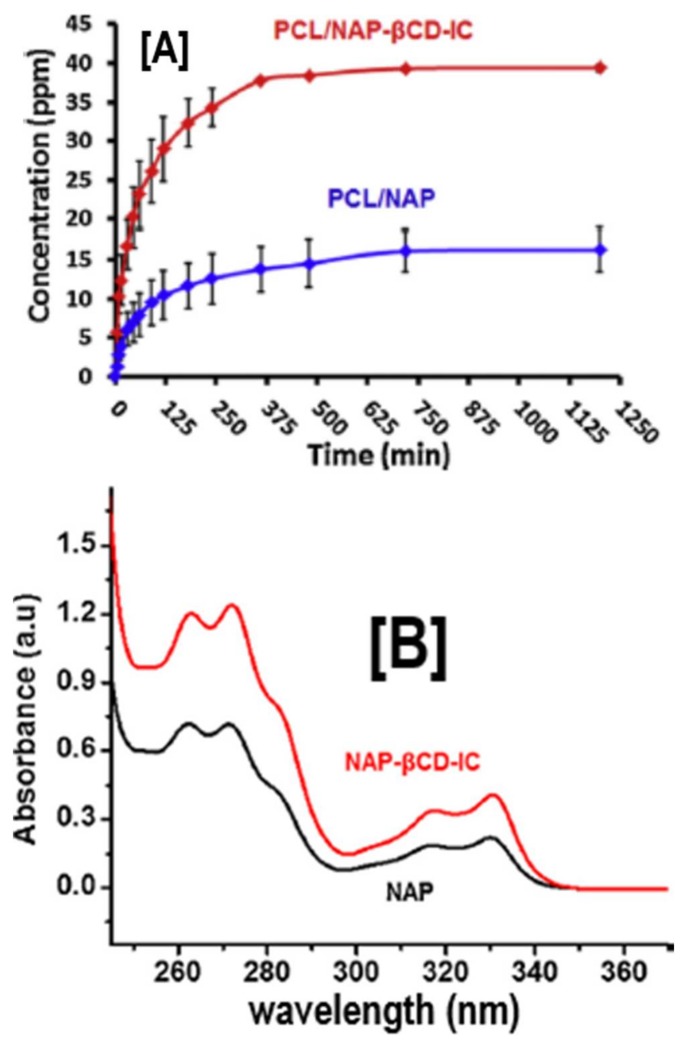
The release and solubility profiles of naproxen (NAP) from PCL and PCL/β-CD-IC nanofibers. The release profile study (**A**) by HPLC revealed significant release of NAP from PCL/NAP-β-CD-IC, compared to the PCL/NAP nanofibers, owing to improved solubility of encapsulated NAP, compared to pristine NAP. These results were further corroborated by solubility analyses (UV-vis) (**B**), which showed significantly enhanced solubility, as observed by increases in the intensity. Images adapted and reproduced from [63]. Copyright Elsevier Ltd., 2014.

In addition to enhancing stability against various environmental factors, enhancing the delivery of drugs, in particular hydrophobic ones, is also another critical factor that has been explored using cyclodextrins as a carrier [58]. Naproxen and sulfisoxazole are hydrophobic drugs that are widely used for relieving pain. Due to their poor solubility, both drugs suffer from slow dissolution and, therefore, are not significantly available at the target site. One common way to improve their solubility and availability is by safely encapsulating them inside CD cavities [59,60,61,62]. When naproxen (NAP) was encapsulated in β-CD-IC cavities via traditional IC formation means, and was subsequently embedded in a PCL nanofibrous matrix, high performance liquid chromatography (HPLC) revealed two-fold improvements in the release rates of NAP, compared to PCL nanofibers with neat NAP (Figure 6A) [63]. This is not particularly surprising considering that UV-vis spectroscopy also showed significantly higher intensities in NAP-β-CD-ICs, compared to NAP alone in water solution (Figure 6B) [63]. Similar to NAP and sulfisoxazole, tetracycline, a biocidal drug, which has poor solubility, has been embedded in PCL nanofibers and encapsulated in β-CD cavities to regenerate periodontal ligaments [64]. Microbiological tests against the commonly found oral pathogens *Aggregatibacter actinomycetemcomitans* (*A.a*) and *Porphyromonas gingivalis* (*P.g*) revealed significantly higher halos of bacterial inhibition against both oral bacteria in PCL nanofibers containing tetracycline/β-CD, compared to PCL nanofibers with (28 ± 4 and 26 ± 3 mm) and without tetracycline (0 and 0 mm) [64]. In addition, PCL nanofibers containing tetracycline/β-CD also showed higher inhibition than chlorhexidine (26 ± 4 vs. 27 ± 3 mm for chlorhexidine), a clinically recommended oral disinfectant [64], illustrating the potential for novel, highly impactful solutions for periodontitis. Ciprofloxacin is yet another hydrophobic biocidal drug whose application has been impaired due to poor solubility and stability [65,66]. By combining ciprofloxacin/β-CD with PCL nanofiber technology, large amounts of ciprofloxacin can be encapsulated, especially when prepared under sonic energy, and subsequently carried in PCL nanofibers [67]. An increase in the released amount of ciprofloxacin *via* this technique has been found in stimulated physiologic environments (pH 7.2) when delivered from β-CD/PCL nanofibers, compared to α-CD/PCL nanofibers [67]. 

Another way to control the solubility of a drug is by utilizing a sandwich of nanofibrous layers containing water soluble and insoluble matrices, as shown recently by Aytac et al. [68]. The authors prepared ICs of sulfisoxazole (SFS) and HP-β-CD, which were subsequently electrospun and carried by hydrophilic, hydroxypropyl cellulose (HPC) nanofibers. As both the carrier polymer and the CDs were highly soluble in water, by sandwiching the HPC/SFS-HP-β-CD nanofibers, release of SFS could be significantly modulated [68]. By employing such a strategy, a slow release of SFS, with the highest amount released over time (~720 min), was realized, demonstrating the existence of several facile strategies to improve/modulate the release of hydrophobic drugs. 

In addition to enhanced retention, solubility, and release of hydrophobic drugs, newer applications of CD functionalized PCL nanofibers are as biocatalysts. It has been recently reported that immobilized biocatalysts on suitable substrates, compared to simple coatings, significantly increase catalytic efficiency and significantly decrease cost, compared to conventional catalysts [69,70,71,72]. More recently, α-amylase, a digestive enzyme immobilized on HP-β-CD, has shown a significant increase in stability, solubility, and enzymatic activity, compared to other routes of administration, such as therapeutic proteins [73]. The large surface to area ratios, high porosity and small pore sizes of nanofibers typically obtained via the electrospinning process give another suitable substrate for biocatalyst immobilization [74]. Recently, electrospun nanofibers possessing pendant CD molecules have been prepared for immobilizing biocatalysts and have been subsequently evaluated for catalytic activities [74]. For instance, catalase, an anti-free radical enzyme, has been successfully immobilized onto PEO nanofibers containing γ-CDs (in complexed and uncomplexed forms), sandwiched between PCL nanofibers [75]. Catalase enzymatic activity results showed the positive influence of CDs on enzyme activity and the stability of the biocatalysts (catalase) over prolonged periods of time [75]. Similar to the catalase enzyme, laccase enzymes immobilized on γ-CD/PCL nanofibers showed higher catalytic activity (96.48 U/mg), compared to enzymes immobilized on PCL nanofibers (without CDs) (23.2 U/mg) or γ-CD/laccase physical mixtures in PCL nanofibers (71.6 U/mg) [76]. In addition to higher enzymatic activity, similar to previous studies on catalase enzyme immobilization, no discernible loss of enzyme activity was observed, indicating a synergistic effect in enhancing catalytic activity by combining polymeric nanofibers and CD-IC formation [76]. 

### 2.2. PCL Nanofibers Functionalized with Cyclodextrin-Large Molecule Inclusion Complexes

The key advantage of CDs is their facile capability to form ICs with both small as well as large molecules. Even though polymer-CD-ICs may not be used directly, as they neither melt nor are soluble in solvents, after coalescing the polymers from the ICs, the coalesced polymers can be processed using conventional means. These coalesced polymers elicit properties that are unique from their neat native samples, making them attractive for a plethora of applications. For example, in the past, we have shown the possibility of synthesizing polymers, such as polystyrene, with well-defined architectures, through a greener route in aqueous solution [77,78]. Coalescing the polymers from the CD cavity results in an intimate blend of immiscible homopolymers or copolymers, as evidenced by single glass transition temperatures, suppressed microphase separation, elevated melting temperatures, and specific interactions observed by DSC and FTIR, respectively [79,80,81,82,83,84,85,86]. In addition, degradation behaviors of these coalesced biodegradable copolymers (PCL-*b-*PLLA) have been observed to be unique, with accelerated enzymatic degradation, in particular during the early phase of degradation, compared to copolymer or physical homopolymer blends [86]. This effect has been observed particularly in the PCL block phase, which could partially be attributed to the degradation environment (*R. arrhizus* in phosphate buffer solution at 37 °C and pH 7) [86]. In the case of homopolymers, even with slow crystallizing polymers, such as polyethylene terephthalate (PET), upon coalescence the ethylene glycol units have been known to adopt a highly extended g ± tg ∓ kink conformation (where g ± are gauche and t is all trans conformations), and PET chains isolated from neighboring chains quickly convert to the fully extended, all-trans crystalline PET conformer [87,88,89]. Even faster crystallizing semi-crystalline polymers, such as PCL, show substantial increases in crystallization temperatures (Δ*T*_c_s) (~25 °C), Young’s modulus (~100%), and hardness [90]. 

While the traditional processing of completely covered polymer chains in CD cavities is not feasible, the processing of polymers can be facilitated if coverage is less than complete. Chains dangling out of the CD cavities are also able to undergo crystallization and melting [91]. In addition, depending on the stoichiometry between the cyclodextrin host and guest polymer, the lengths of dangling polymer chains and their molecular weights, crystallizability (*T*_c_s and *T*_m_s) increases significantly, irrespective of the crystalline nature of the dangling polymer [50,92,93]. Besides improvements in thermal properties, the additions of such non-stoichiometric ICs to bulk polymers have also resulted in significant increases in elastic modulus values and tensile strength, with drastic reductions in the loss tangent and elongation, indicating significant improvements in the stiffness and elasticity of the composites [94]. This is particularly interesting, as the evidenced improvements occurred over a wide range of stoichiometries (the more dangling chains the better) and temperatures (−50 to 50 °C) [94].

**Figure 7:**
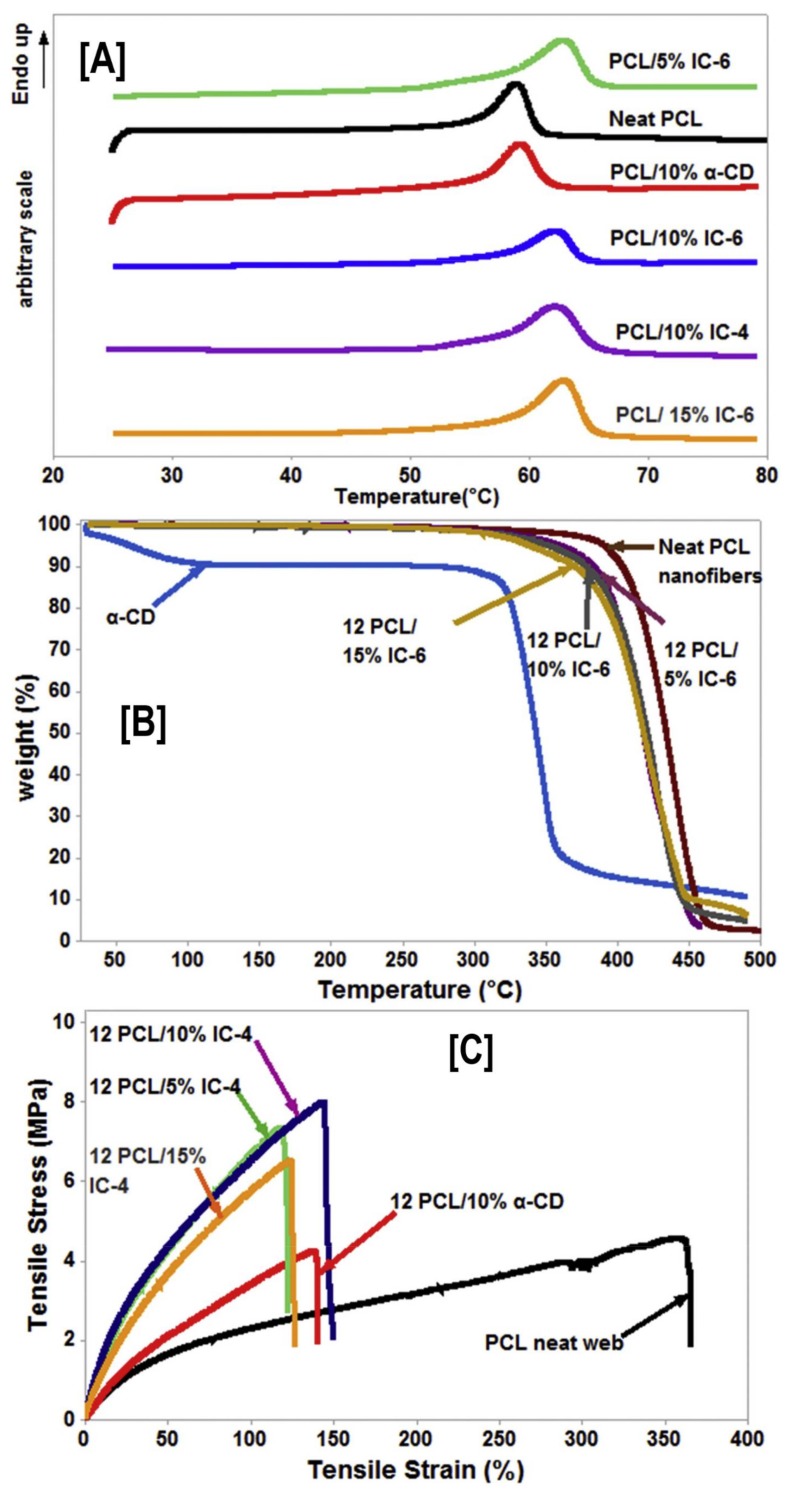
Structure–property relationships (thermal and mechanical), due to the presence of intact non-stoichiometric inclusion complexes (n-s PCL-α-CD-IC) in PCL nanofibers. The presence of intact (n-s PCL-α-CD-IC) caused increases (~5 °C) in PCL melting temperature (**A**), irrespective of the stoichiometric ratio of the IC or the wt % loading of those ICs in the nanofibers. Likewise, the degradation temperature of the α-CD phase significantly increased in those composites, indicating the presence of α-CD in complexed state (**B**). Finally, intact ICs caused changes in the mechanical behavior (increased modulus value and decreased elongation) of the composites. Images adapted with permission from [95]. Copyright Elsevier Ltd., 2015.

Recently, our group took advantage of non-stoichiometric inclusion complexes (PCL-α-CD-ICs) by using them as nucleating agents for neat bulk PCL nanofibers [95], as well as synthesizing pseudorotaxane nanofibers (PCL-α-CD-ICs) without a polymeric carrier [96,97]. In the first instance, three ICs were synthesized with stoichiometries of 1:1 (complete coverage), 4:1, and 6:1 (PCL:CD molar ratio) [95]. The as-synthesized ICs were characterized by ^1^H-NMR (for stoichiometry) [98], wide angle X-ray diffraction, FTIR, and DSC, respectively [95]. The ICs (5, 10, and 15% wt loading) were then added to PCL solutions (in 60:40 chloroform/*N*,*N* dimethyl formamide (CFM/DMF)), and were electrospun into nanofibers (PCL/PCL-α-CD-ICs). In the first heating cycle, DSC analyses showed elevated melting temperatures (*T*_m_), and in TGA analyses, higher degradation temperatures of the composites were observed, indicating the presence of intact ICs in the composite nanofibers (Figure 7A,B) [95]. More interestingly, quasi-static tensile testing of PCL/PCL-α-CD-ICs indicated significant increases in modulus values, with corresponding decreases in elongation values at all wt % IC loadings, compared to control nanofibers (PCL and PCL/α-CD) [95]. Within the wt % loading and stoichiometry evaluated, 10% IC-6 (6:1 stoichiometric ratio) loading resulted in a high tensile modulus value (15.2 ± 5.0 MPa) and low elongation (170 ± 40%), compared to 2.6 ± 0.9 MPa and 390 ± 70%, respectively, observed for neat PCL nanofibers [95]. 

An identical trend was also observed in IC-4 (4:1 stoichiometric ratio) nucleated PCL composites, with a high tensile modulus value (10.2 ± 1.2 MPa) and low elongation (170 ± 20%) observed for composites containing 10% IC-4 [95] (Figure 7C), indicating the necessity of optimal stoichiometry, as well as the wt % loading for improving the mechanical properties of PCL nanofibers for potential applications in tissue engineering. 

By employing a similar approach, our group also fabricated intact PCL-α-CD-IC nanofibers with coverages ranging from 3% to 12% from CFM solution, facilitated by the addition of benzyl triethylammonium chloride salt (BTEAC) to increase solution conductivity [96]. Preliminary FTIR analyses provided qualitative assessment of the varying stoichiometries in the nanofibers by decreasing carbonyl peaks (bending vibration at 1027 cm^−1^) and increasing hydroxyl peaks (3351 cm^−1^), with increases in the coverage of PCL chains by α-CD [96]. Similar to the previous study that reported PCL/PCL-α-CD-IC composites, increases in thermal stability and the channel-like crystal structure of IC nanofibers were observed by TGA and WAXD analyses, respectively, and provided conclusive evidence as to the presence of intact rotaxanated structures [96]. Finally, just like the PCL/PCL-α-CD-IC study, mechanical testing showed a substantial increase in modulus values (16.0 ± 4.9 vs. 8.0 ± 1.7 MPa for neat PCL nanofibers) [96]. 

To further understand the structure–property relationships that exist in liquid (polymer solutions) and solid phases (nanofibers), a subsequent study was carried out to evaluate the rheology of solutions, and 2D-WAXD, selected area electron diffraction (SAED), and dynamic mechanical analyses were performed on the nanofibers [97]. The frequency sweep of PCL and pseudorotaxane solutions showed significant differences in terms of elastic and loss moduli and cross-over patterns within the studied frequency ranges. While the neat PCL solution had both lower elastic, as well as loss moduli, the PCL solutions also displayed several cross over points, indicating the separation of PCL chains from one another, as would be expected for viscoelastic materials [97]. 

**Figure 8:**
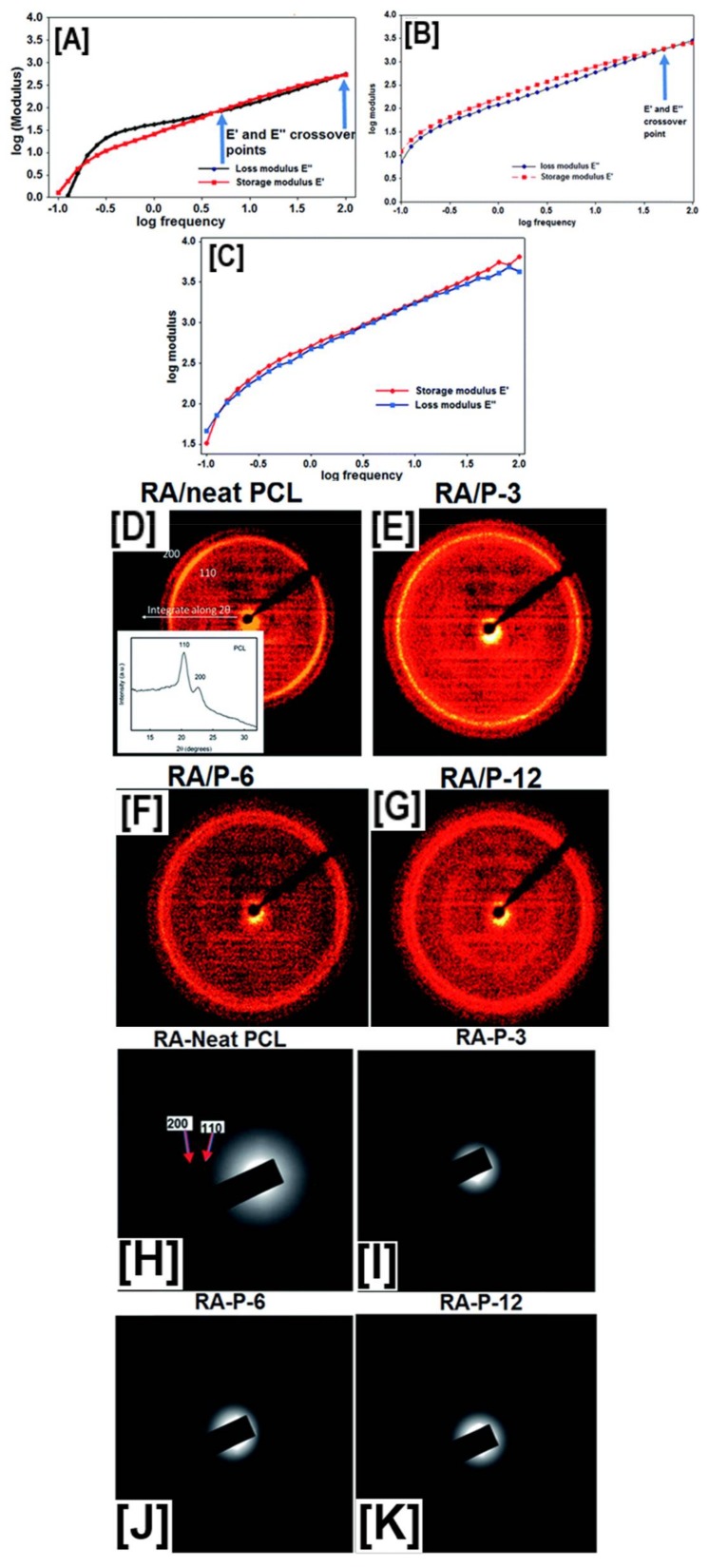
Establishing structure property relationships between pseudorotaxane (PpR) solution rheology and the resultant molecular arrangement of PpR in the nanofibers. Solution rheology of neat PCL solutions at higher concentrations (12%) showed low modulus values (elastic and loss) with multiple cross-over points, typical of viscoelastic material. On the other hand, PpR solutions showed frequent cross-over points, indicating a Rouse–Zimm effect (**A**–**C**). However, both 2D-wide angle X-ray diffraction (**D**–**G**) and selected area electron diffraction analyses (**H**–**K**) showed the presence of crystalline domains in both randomly aligned, as well as aligned PCL nanofibers, while no such peaks were evidently observed in PpR nanofibers. Images adapted and reproduced with permission from [97]. Copyright The Royal Society of America 2016.

In P-3 solutions (3% coverage by CDs), a solitary cross-over point was observed at higher frequencies, indicating longer relaxation times for the excluded portions of the PCL chains. In P-12 solutions (12% coverage by CDs), this cross-over was observed at even higher frequencies, leading to Rouse–Zimm-like behavior, which would be typically expected of unfettered polymer chains in dilute solutions, leading to significant increases in the elastic moduli (Figure 8B,C) [97]. Further 2D-WAXD and SAED experiments (Figure 8D–K) demonstrated the presence of crystalline domains in control PCL nanofibers (both aligned and random) corresponding to the (110) and (200) reflections, while all the pseudorotaxane nanofibers showed an absence of reflections, indicating the randomness of the crystal lattice arrangement along the fiber axis [97]. Contrastingly, both quasi-static tensile testing, as well as dynamic mechanical analyses (DMA), provided further insights into the deformation behavior of the composite and control nanofibers. With an increase in the coverage of PCL chains by CDs, linear trends of increasing modulus values (14 ± 5 vs. 16 ± 6 vs. 29 ± 5 MPa for P-3, P-6, and P-12, respectively) and decreasing elongation (103 ± 34 vs. 62 ± 12% for P-3, and P-12, respectively) were observed [97]. Just like quasi-static tensile testing, DMA analyses also showed a similar trend, with high elastic and loss moduli values observed for P-12 > P-6 > P-3 > neat PCL nanofibers, at most studied temperatures, with the key difference being a gradual decrease in the moduli values, compared to a sharp decrease observed for neat PCL nanofibers, indicating a strong intermolecular interaction between pseudorotaxane nanofibers [97].

One of the foremost advantages of polymer coverage by CDs is their potential ability to immobilize proteins/growth factors/biomolecules, owing to the presence of abundant hydroxyl groups present on the external rims of the CDs [11,99]. This is typically accomplished by a variety of post-modification reactions on hydroxyl groups, facilitating intermolecular interactions, such as ionic interactions [100,101] and click chemistries [102], potentially resulting in the immobilization of bioactive molecules. Although our studies did not focus on further development of the functionalized PCL, Elisseeff [103] and Schlatter [104,105] have reported various bioactive molecule immobilization on PCL-α-CD-IC nanofibers. 

**Figure 9:**
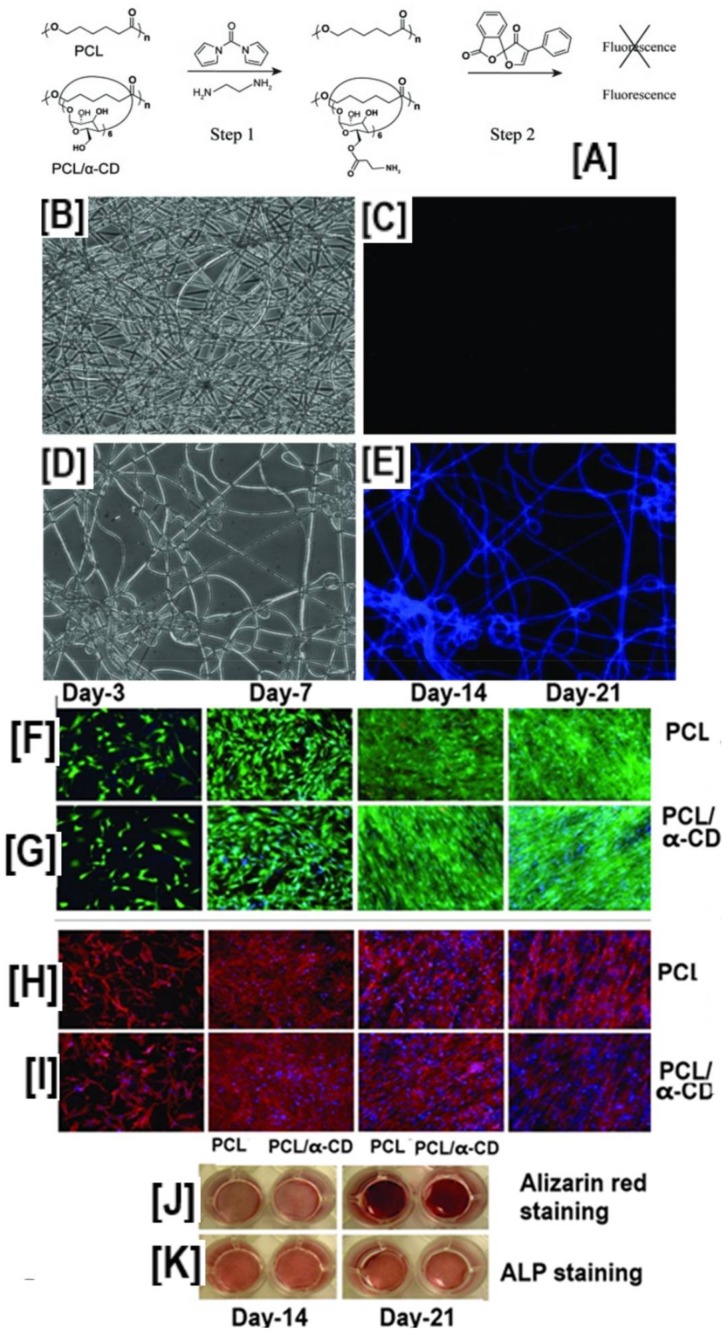
Schematic illustrating the immobilization of fluorescent molecules and the effect of hydroxyl groups of cyclodextrins on the osteogenic differentiation of human adipose-derived stem cells. The Neat PCL does not have surface epitopes facilitating immobilization of bioactive molecules. Conversely, the pseudorotaxanes contain abundant hydroxyl groups, facilitating the immobilization of bioactive molecules, and a representative fluorescent molecule (fluorescamine) (**A**), whose presence was monitored by fluorescent spectroscopy (**B**–**E**). Although immobilization of active biomolecule was possible only in scaffolds containing CDs, live-dead cell assay and F-actin assay showed no statistical significance in the cell viability at any of the time point studied (**F**–**I**) In addition to facilitating the immobilization of bioactive molecules, PCL/α-CD nanofibers without immobilization of biomolecules also facilitated the osteogenic differentiation of human adipose derived stem cells (h-ADSCs), although the osteogenic marker levels showing similarities in the expression levels between the study groups were the same (**J,K**). Images adapted and reproduced with permission from [103]. Copyright Springer 2012.

Elisseeff’s research group, in proof-of-concept experiments, showed the conjugation of fluorescamine onto PCL-α-CD-IC nanofibers upon activation of the hydroxyl groups by *N*,*N*′-carbonyldiimidazole (CDI) (Figure 9A–E) [103]. 

Even though further studies on immobilizing bioactive molecules were not conducted, unconjugated PCL-α-CD-IC nanofibers were evaluated for their potential role in bone tissue engineering. Cell viability/proliferation, cytoskeleton formation, calcium mineralization, and gene expression assays, were carried out on PCL and PCL-α-CD-IC nanofiber scaffolds seeded with human adipose-derived stem cells (h-ADSCs) [103]. Despite cell viability or mineralization assays not showing significant differences over a 21-day period (cell viability: 96.5 ± 2.5% and 97.1 ± 1.5% live cells for PCL and PCL-α-CD-IC nanofibers) (Figure 9F,G), gene expression levels of osteogenic markers such as RunX2, osteopontin (OPN), COL1A1, COL3A1, COLXA1, and hydroxyproline levels were upregulated in PCL-α-CD-IC scaffolds compared to PCL nanofibers, indicating the potential application of PCL-α-CD-IC scaffolds in bone tissue engineering [103]. 

**Figure 10:**
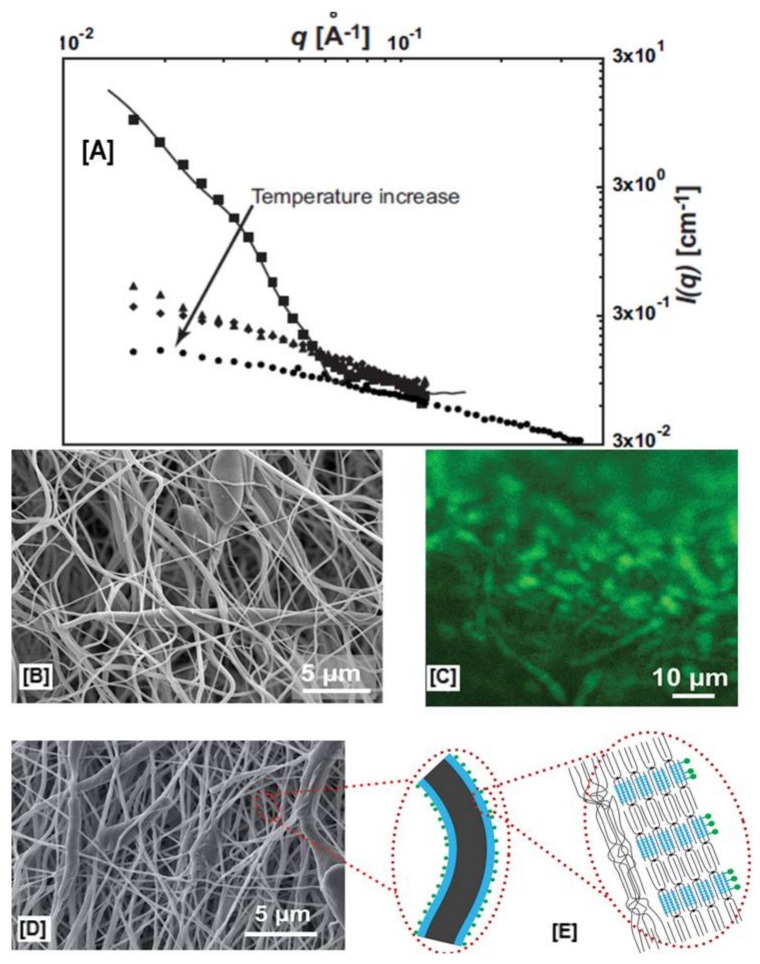
Temperature-dependent stability of pseudorotaxanes (PpR) using DMSO and an electron micrograph of electrospun core–shell nanofibers with PpR in the shell region, which was subsequently conjugated with FITC. The SANS analyses of the PpR (shell region) in DMSO showed decreases in scattering intensity, indicating dethreading of PpRs into α-CD and PCL phases. Whereas at 35 °C, higher scattering intensity indicated the presence of a PpR structure, and in addition, a parallelepiped model was developed which showed CD assemblies approximating 11 α-CDs in length and 20 α-CDs in width (**A**). Additionally, conjugation of fluorescamine was only possible onto hydroxyl groups present in PpR (**B**–**E**), whereas in neat PCL nanofibers, conjugation was not possible. Images adapted and reproduced with permission from [104]. Copyright John Wiley and Sons 2015.

As we have shown in our PCL-α-CD-IC electrospinning experiments [96,97], the addition of DMF or dimethyl sulfoxide (DMSO) leads to the destabilization of the rotaxanated structure, leading to the de-threading of PCL from CD cavities. We overcame this difficulty by electrospinning pseudorotaxanes (PpR) from CFM and improved solution conductivity with the addition of BTEAC salt. Another easier method was reported by Schlatter’s research group, who reported on the fabrication of core–shell nanofibers comprised of four branched star-PpR or mikto-arm PpR for shell and neat PCL for core regions, respectively [104,105]. Small angle neutron scattering measurements (SANS) showed temperature-dependent shell stability/core instability of PpR in DMSO, based on which stable PpR solution was prepared for electrospinning (35 °C) (Figure 10A). WAXD analyses of PCL/PpR nanofibers (10:2 and 10:3 ratios) showed a 2θ = 20° peak, indicating the presence of intact rotaxane crystals in the coaxial spun nanofibers [104]. The intact PpR was further taken advantage of by immobilizing fluorescein isothiocyanate (FITC) onto star PpR *via* immersing the scaffolds in acetonitrile, a water (1:1) solution containing FITC [104]. Interestingly, confocal microscopy experiments showed fluorescence in core–shell fibers containing star-PpR in the shell region (Figure 10B,C), whereas in the core–shell nanofibers containing linear PpR, such a fluorescent effect was not observed due to the dethreading and subsequent leaching of CDs [104]. 

Using a similar approach, FITC was immobilized onto both star-PpR, as well as mikto-arm PpR (two of the four ends were covered by CDs), with both nanofibers showing fluorescent activity. However, some damage to the mikto-arm PpR containing nanofibers was observed, indicating some degree of PpR structure destabilization in nanofibers by DMSO [105]. In addition to FITC, a red fluorescent molecule, Megastokes 673 was immobilized onto PCL, star-, and mikto-arm PpRs, with no fluorescent activity observed in PCL nanofibers, again demonstrating the necessity of functional groups on the nanofibers surface for bioconjugation [105]. 

### 2.3. Cyclodextrins or Inclusion Complex Functionalized Poly(lactic acid) Nanofibers

Just like PCL, poly(lactic acid) (PLA) belongs to the class of aliphatic polyesters, also known as α-hydroxy esters, that are renowned for their excellent bioresorbable and biocompatible characteristics, making them suitable for pharmaceutical and biomedical implants. Unlike PCL, which is a fast crystallizing polymer, PLA is a slow crystallizing, semi-crystalline polymer with crystallinity, melting, and glass transition temperature values ranging from 40–50 °C, 55–80 °C, and 170–180 °C, respectively [4]. Another difference lies in the chirality of PLA, enabling PLA to exist in three enantiomeric states, namely l-Lactide (PLLA), d-Lactide (PDLA), and *meso*-lactide, with PLLA and PDLA commonly used as biomedical implants, in particular in orthopaedic and dental implants [4]. To further improve degradation characteristics, material properties (for example, thermal and mechanical), and processing behavior, lactic acid monomers are copolymerized with various monomers, including other aliphatic polyester monomers such as ε-caprolactone and glycolic acid, or with monomers such as ethylene glycol and glutamic acid, making PLA a versatile biopolymer [4]. 

Even though some of the surface properties of these bulk copolymers were enhanced, uncontrolled detrimental effects were observed on other properties, necessitating the development of a newer “softer” approach in functionalizing PLA-based materials. Just like PCL, PLA-based nanofibers have been functionalized with cyclodextrins or their inclusion complexes, leading to the development of materials suitable for anti-cancer therapies, modulating drug release, anti-microbial packaging, and wound dressing [106]. 

In one study, slow degradation characteristics (compared to hydrophilic polymers) of poly(lactic-*co*-glycolic acid) (PLGA) were taken advantage of by forming sequential layers of hydrophilic PVA nanofibers containing haloperidol, encapsulated in randomly methylated β-CD (RM-β-CD) cavities, resulting in the slow, controlled released of haloperidol [107]. While the release of haloperidol was also low from PVA nanofibers without β-CD encapsulation, the drug release was instantaneous and the fibers dissolved rapidly when encapsulated in RM-β-CD cavities. On the other hand, in sequential layers containing PLGA nanofibrous layers, a more controlled release of haloperidol over several days was observed, with some initial burst of the drug, along with improved sensitivity towards heat and light, and low water solubility [107]. 

While this study evaluated the release of haloperidol from single- or multi-layered nanofibrous films, structural changes, which determine the efficacy of the drug, were not evaluated. Xie et al., for instance, evaluated both the release rates, as well as the form of released hydroxycamptothecin (HCPT), an active drug prescribed for treating malignant tumors, from poly(ethylene glycol)–poly(dl-lactic acid) (PELA) copolymer nanofibers [108]. With the addition of HCPT in encapsulated form in 2-hydroxypropyl-β-CD (HP-β-CD), greater release of HCPT from HCPT/PELA fibers was observed, depending on the HP-β-CD concentration (5% > 2.5% > 1.5%). For example, at HP-β-CD concentrations of 1.5% and 2.5%, respectively, 50.5 ± 3.8% and 58.2 ± 2.0% of HCPT was released from the PELA fibers after 20-days of inoculation, attributed to the enhancement of drug dissolution by hydrophilic HP-β-CD [108]. Also, with the addition of HCPT in complexed form, the half-life of the active drug form (lactone) improved from 24 to 40 mins in physiologic conditions, and in addition, the half maximal inhibitory concentration (IC_50_) of the drug improved dramatically, by 7 times compared to free HCPT [108], indicating the therapeutic potential of drug-CD embedded PLA nanofibers. 

**Figure 11:**
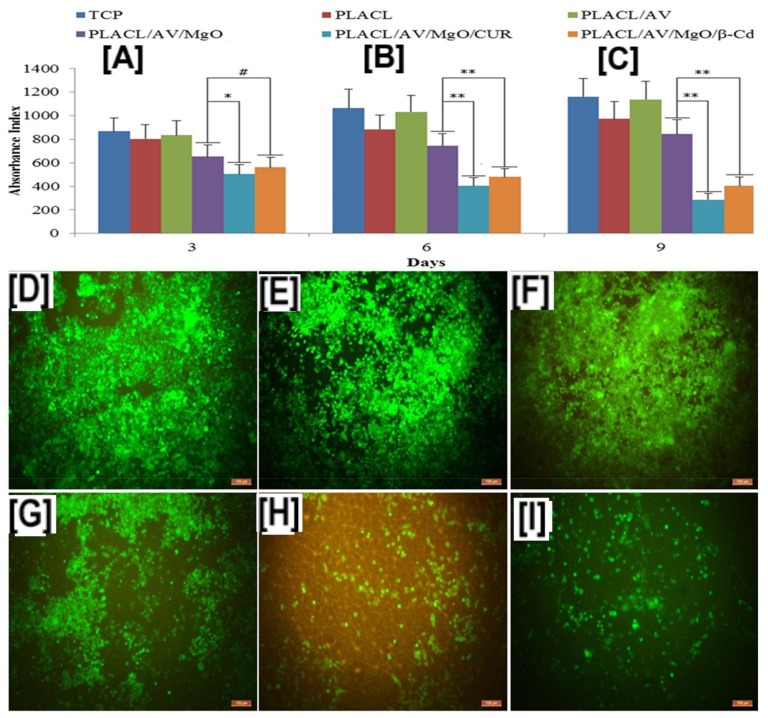
Proliferation and cell densities of Michigan Cancer Foundation-7 (MCF-7) cells on poly(lactide-*co*-caprolactone) (PLCL or PLACL) scaffolds and PLCL scaffolds containing aloe vera (AV), magnesium oxide (MgO), and free curcumin (CUR), or encapsulated in β-CD. The addition of AV alone did not cause MCF-7 cell death at all studied time points; however, the addition of CUR, in either free or encapsulated form, resulted in significant cell death of the proliferating MCF-7 cells (**A**). These results were further corroborated by laser confocal scanning microscopy measurements, which showed low cell densities in scaffolds containing CUR and MgO/CUR (11 B: (**E**,**F**)). Images adapted and reproduced with permission from [109]. Copyright Elsevier Ltd., 2017.

Similar to the study of HCPT for treating cancer, curcumin (CUR) encapsulated in β-CD carried by poly(lactide-*co*-caprolactone) PLCL nanofibers, in conjunction with other biologics, such as aloe vera (AV) and magnesium oxide (MgO) nanoparticles, have been evaluated for treating breast cancer [109]. In vitro proliferation evaluation of the composite fibers of breast cancer cell line, MCF-7 (Michigan Cancer Foundation-7), by MTS assay (3-(4,5-dimethylthiazol-2-yl)-5-(3-carboxymethoxyphenyl)-2-(4-sulfophenyl)-2H-tetrazolium, inner salt) showed a significant reduction in cell numbers (*p* < 0.01) after 6- and 9-days, with either free or encapsulated CUR (PLCL/AV/MgO/CUR and PLCL/AV/MgO/CUR/β-CD scaffolds) (Figure 11B,C) [109]. These results were corroborated by laser confocal scanning microscopy images of fluorescent dye 5-chloromethylfluoresceindiacetate (CMFDA) (chloromethyl derivatives)-labelled MCF-7 cells, which showed decreased cell densities in scaffolds containing free or encapsulated curcumin, compared to the scaffolds that did not contain curcumin (PLCL/AV, PLCL/MgO, and controls) (Figure 11D–G) [109]. In addition, CUR-containing scaffolds (PLCL/AV/MgO/CUR and PLCL/AV/MgO/CUR/β-CD) showed a drastic reduction in collagen secretion, indicating poor proliferation of MCF-7 cells, due to the presence of CUR/β-CD, and showing a synergistic effect of CD/drugs delivered via PLA-based nanofibers (Figure 11H,I) [109]. 

Given the fact that release of drugs or other small molecule guests are significantly enhanced with encapsulation in CD cavities, drug release can also be controlled by simply encapsulating the complex in the core region of PLA nanofibers. Using such a facile strategy, CUR/HP-β-CD ICs were safely embedded in the core region, with the PLA forming the shell region of the coaxial nanofibers [110]. Although the rate of CUR dissolution from core–shell nanofibers was slow, the total release of CUR from core–shell nanofibers was significantly higher at simulated physiologic conditions (pH 1 and 7.4). In addition, release of CUR from PLA nanofibers and core–shell nanofibers were evaluated in methanol and methanol–water (1:1), which showed higher release of CUR from PLA nanofibers into the methanol solution [110]. On the other hand, CUR released from core–shell nanofibers was higher in methanol–water, owing to improved dissolution in aqueous solution, due to the presence of HP-β-CD [110]. In accordance with the release study, antioxidant activity of CUR in methanol and methanol–water, also showed a similar trend, with PLA/CUR nanofibers and core–shell nanofibers, showing improved activity in methanol and methanol–water solutions, respectively [110]. 

**Figure 12:**
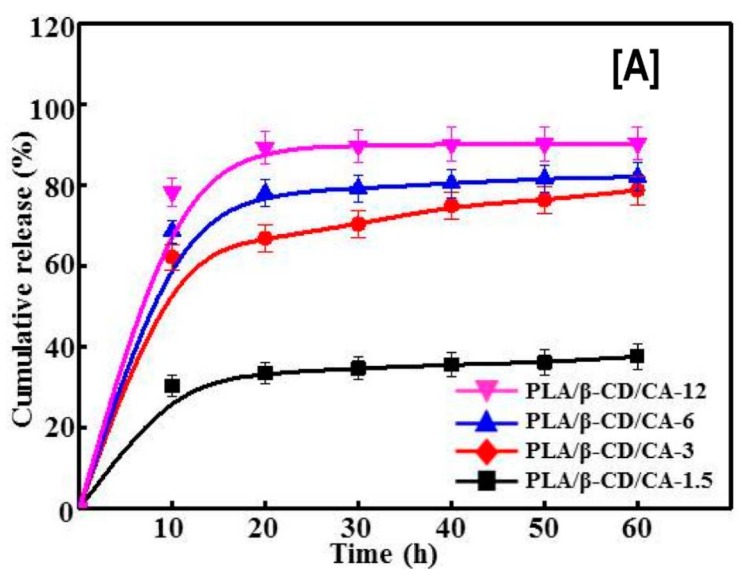

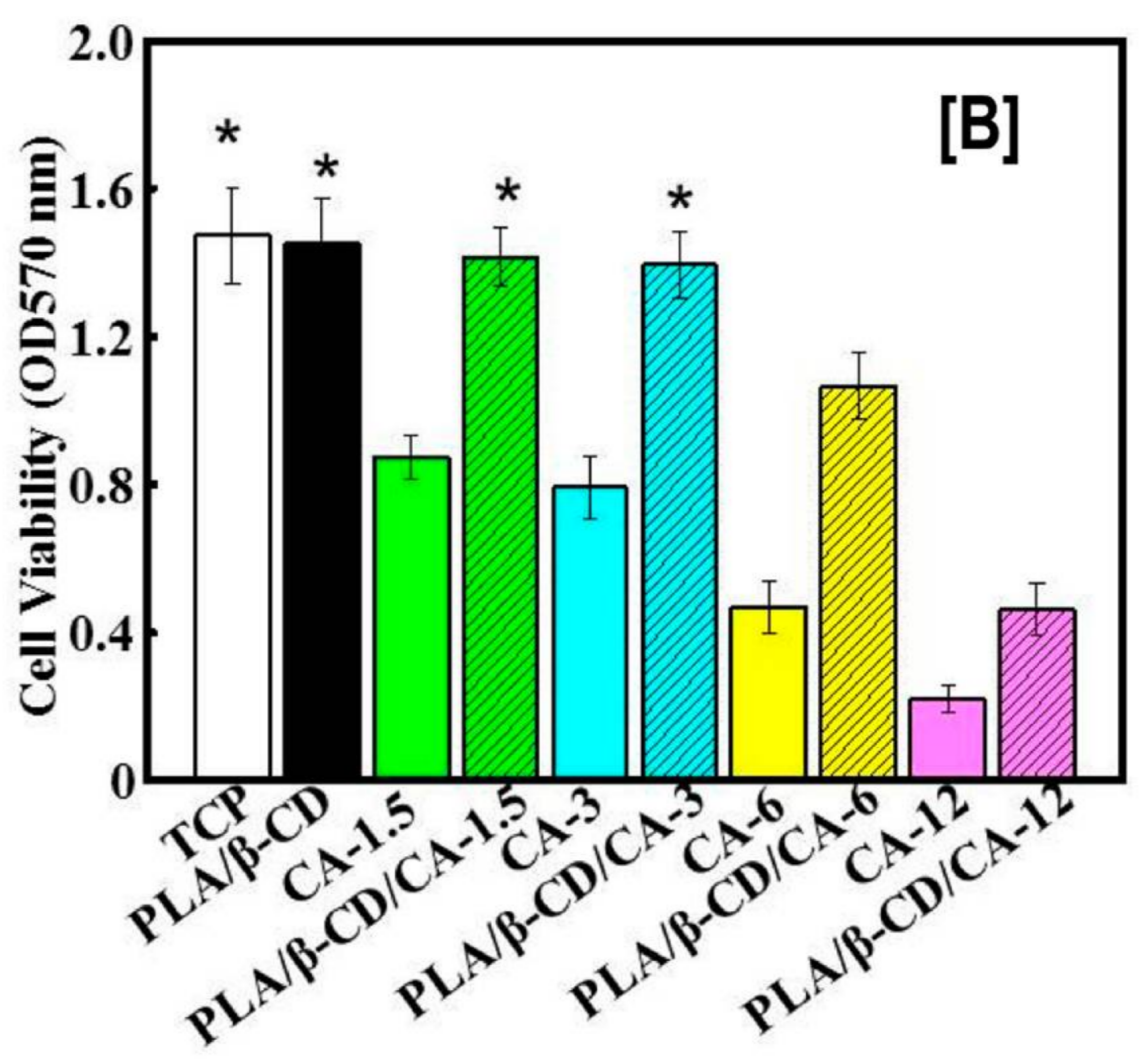
The effects of poly(lactic acid) nanofibers containing β-CD/cinnamaldehyde (CA) inclusion complex on the cumulative release of CA and cell viability of human skin fibroblasts. The cumulative release of CA depended on the β-CD/CA concentration, i.e., the higher the concentration, the higher the release rate of CA, possibly owing to improved dissolution of CA from β-CD complex (**A**). In comparison, low cell densities of *S. aureus* and *E. coli* were observed with increasing β-CD/CA complex concentrations. Similar trends were also seen with the proliferation of human skin fibroblasts cells, when exposed to CA containing PLA nanofibers or free CA (**B**). Images adapted and reproduced with permission from [111] Copyright Liu et al., 2017.

Just like the delivery (rapid or controlled) of hydrophobic anti-cancer drugs is critical for treating cancer, improving the efficiency of antimicrobial agents, such as cinnamaldehyde (CA), is critical for treating wounds. Recently, CA encapsulated in β-CDs delivered by PLA nanofibers was evaluated for bactericidal effects against *Escherichia coli* (*E. coli*) and *Staphylococcus aureus* (*S*. *aureus*), and for cytocompability against CCC-HSF-1 human skin fibroblasts (HSFs) [111]. With the addition of CA-β-CDs in PLA, concentration dependent effects of CA-β-CDs on average fiber diameter (the higher the loading, the higher the fiber diameter), were observed. While this trend was reversed for tensile modulus values and surface hydrophilicity of the nanofibers (the higher the loading, the lower the modulus value and surface hydrophilicity) [111]. In addition, similar to the release rates observed in anti-cancer agents, the cumulative release rate of CA also depended on the concentration of CA-β-CDs (12% > 6% > 3% > 1.5%) used in the nanofiber formulation (Figure 12A). Notwithstanding the fiber diameters or the enhanced release rate of CA, a concentration (of CA-β-CD) independent effect on cell death of *S*. *aureus* and *E*. *coli* with the addition of CA-β-CD was observed. In other words, the addition of CA-β-CD resulted in poor viability (>95% cell death) of *S*. *aureus* and *E*. *coli* at all studied times (20, 40, and 60 h) [111]. Another advantage of utilizing CD-ICs over free CAs was observed in cell viability (Figure 12B), where the toxicity of CA was significantly decreased by its encapsulation, indicating the possibility of modulating drug response against bacteria and physiologic cells by varying the concentration of ICs [111]. 

Exploding population growth requires increased food production, as well as the prevention of food spoilage due to microbial action. For these reasons, there is an immediate need to curtail the growth of food borne microbes and, thereby, extend the shelf life of food products, without significant loss of nutritional and sensory characteristics. In addition, prevention of active ingredients against volatility, light, heat, and air, are other factors that need to be actively addressed, such as through encapsulation inside CD cavities. Several active ingredients, such as gallic acid, triclosan, and essential oils (cinnamon), have been investigated, in conjunction with PLA nanofibers, for their capability preventing microbial growth. For instance, triclosan (TR) has been encapsulated in β- and γ-CDs (not possible in α-CD cavity), which were subsequently electrospun with a polymeric carrier (PLA), resulting in nanofibers with fiber sizes ranging from 640 ± 480 and 940 ± 500 nm for PLA/TR/β-CD-IC and PLA/TR/γ-CD-IC nanofibers, respectively [112]. Successful complexation was confirmed by DSC and TGA analyses, which showed an absence of TR melting (typically seen at ~60 °C) and the enhanced thermal stability of TR, indicating successful IC formation. An antibacterial test of the electrospun nanowebs (PLA/TR, PLA/TR/β-CD-IC, and PLA/TR/γ-CD-IC) showed significant increases in the inhibition zones against *S. aureus* and *E. coli* for the PLA/TR/CD system over the PLA/TR system [112]. In particular, the TR/β-CD-IC system showed significant inhibition zones (3.50 ± 0.00 and 3.7 ± 0.15 cm) against *S. aureus* and *E. coli*, compared to the PLA/TR/γ-CD-IC system, which showed inhibition zones of 2.90 ± 0.17 and 3.3 ± 0.00 cm, respectively, that in turn, were significantly higher than PLA/TR (2.83 ± 0.28 and 3.0 ± 0.20 cm) [112]. 

**Figure 13:**
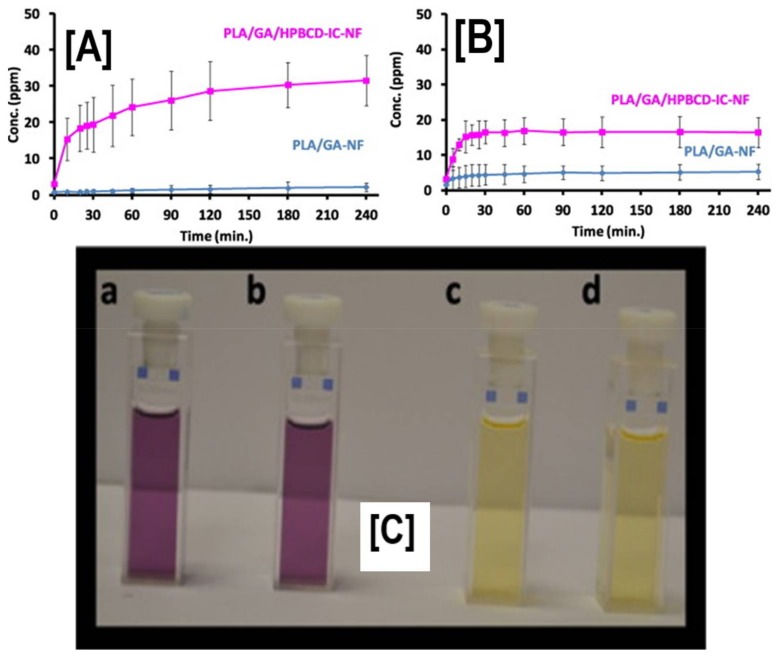
Total cumulative release and antioxidant characteristics of gallic acid (GA) released from PLA/GA-HP-β-CD nanofibers. The total cumulative release of GA from PLA/GA-HP-β-CD nanofibers was always higher in water (**A**) and 10% ethanol solutions (**B**), than those released from PLA/GA nanofibers, owing to the enhanced dissolution characteristics of GA/HP-β-CD compared to free GA. In addition, the DPPH assay showed that the presence of GA in both PLA/GA, as well as PLA/GA-HP-β-CD nanofibers, retained its antioxidant characteristics (**C**). Images adapted and reproduced with permission from [114]. Copyright Elsevier Ltd., 2016.

Unlike TR, which is a synthetic compound, due to health and environmental considerations, antimicrobial agents from natural sources, such as gallic acid and cinnamon oil (CEO), are also being actively investigated for their antimicrobial and antioxidant characteristics in food packaging. Similar to PLA/TR/CD systems, PLA/CEO showed enhanced inhibition against *S. aureus* and *E. coli*, with quantitative evaluation demonstrating a drastic reduction in the viability of microbes for up to 9 days [113]. In addition to antimicrobial activity, some of these agents are also antioxidants that can scavenge free radicals from the system. Gallic acid (GA), an antimicrobial/antioxidant agent derived from plants and herbs, has been encapsulated in HP-β-CD, and subsequently fabricated into bead-free PLA/GA-HP-β-CD nanofibers [114]. While the loading was obviously low in PLA/GA-HP-β-CD nanofibers (75 ± 0.7%) compared to PLA/GA nanofibers (80 ± 0.7%), the cumulative release of GA from these systems was significantly higher in ethanol solutions (10% and 95%) (Figure 13A). In addition, GA from PLA/GA-HP-β-CD nanofibers retained excellent antioxidant characteristics, as was evidenced by DPPH (2,2-diphenyl-1-picrylhydrazyl) assay (Figure 13C) [114]. 

## 3. Future Outlook

The aliphatic polyesters discussed in this review are well-studied materials for biomedical applications and are known for their excellent biocompatible and bioresorbable characteristics. Despite these features, both PCL and PLA are known for their hydrophobicity, rendering their utility in pharmaceutical applications, where the controlled or rapid delivery of drugs is desirable. In addition, despite being a fast crystallizing, semi-crystalline material, PCL suffers from poor mechanical properties (low modulus value, strength, and high elongation). Modulating small molecule delivery/capture using a facile approach significantly increases its appeal for these applications. 

Although CDs were originally developed predominantly for the rapid delivery of drugs, which are mostly hydrophobic, CDs have also been utilized to improve the properties of polymers in several ways, for example, by reorganizing polymer chains [115]. As seen from this review, aliphatic polyesters can be combined with CDs or CD-ICs in numerous ways and can be fabricated into nanofibers; however, significant research needs to be carried out. For example, to date, no in vivo studies have been carried out to explore the safety and efficacy of aliphatic polyester/CD or IC nanofibers. In addition, almost all studies deal with nanofibers in non-woven mats; however, additional processing, such as textile braiding, would further improve the prospects of these fibers for biomedical applications. 

There is a lack of literature detailing the use of other aliphatic polyesters, for example, poly(glycolic acid) and poly(lactic-*co*-glycolic) acid, which are well-known as suture and drug delivery materials, owing to their fast degradation nature. Not only could combining these polymers with CD-based strategies lead to novel materials with improved properties, but based on further in vivo experiments, it is quite likely that application-specific or tailorable materials could be developed for a wide range of biomedical applications. In addition, these polymers are approved as “generally regarded as safe” (GRAS) by the Food and Drug Administration for a variety of applications. In combination with the GRAS status of CDs, this is expected to accelerate the utility of these materials in biomedical applications. 

## 4. Conclusions

We have presented some of the combinations of aliphatic polyester nanofibers functionalized with cyclodextrins or their inclusion complexes, that have been reported predominantly in the past five years. A striking observation is that various strategies are now available to fabricate polymeric nanofibers containing free cyclodextrins or their small molecule inclusion complexes or even large molecule (polymer) inclusion complexes, with or without even a polymer carrier. In addition, if added cyclodextrins encapsulate guest molecules, it is now possible to tailor/modulate release rates by using, for example, multilayered sheets or core–shell structures. Just like in solid powder form, encapsulating results in rapid dissolution of hydrophobic molecules in aqueous solution (and possibly into physiologic tissues in vivo). Additionally, the structures of the drug molecules remain intact, meaning they retain their pharmacologic, antimicrobial, and antioxidant effects, which may not be possible without a cyclodextrin carrier. 

## Abbreviations

*A.a*	*Aggregatibacter actinomycetemcomitans*
AV	Aloe vera
BTEAC	Benzyl triethylammonium chloride salt
CA	Cinnamaldehyde
CDs	Cyclodextrins
CDI	*N*,*N*′-carbonyldiimidazole
CD-ICs	CD-inclusion complexes
CFM	Chloroform
CMFDA	5-chloromethylfluoresceindiacetate
CEO	Cinnamon essential oil
CUR	Curcumin
DMA	Dynamic mechanical analyses
DMSO	Dimethyl sulfoxide
DMF	Dimethyl formamide
DPPH	2,2-diphenyl-1-picrylhydrazyl
DSC	Differential scanning calorimetry
*E. coli*	*Escherichia coli*
FITC	Fluorescein isothiocyanate
GA	Gallic acid
hADSCs	Human adipose-derived stem cells
HP-β-CD	2-hydroxypropyl-β-CD
HPC	Hydroxypropyl cellulose
HPLC	High performance liquid chromatography
HCPT	Hydroxycamptothecin
HSF	Human skin fibroblasts
IC_50_	Half maximal inhibitory concentration
MCF-7	Michigan Cancer Foundation-7
MgO	Magnesium oxide
NAP	Naproxen
OPN	Osteopontin
PCL	Poly(ε-caprolactone)
PDLA	Poly(d-lactic acid)
PELA	Poly(ethylene glycol)–poly(dl-lactic acid)
*P.g*	*Porphyromonas gingivalis*
PLCL	Poly(lactide-*co*-caprolactone)
PLLA	Poly(l-lactic acid)
PpR	Pseudorotaxanes
PLA	Poly lactic acid
RM-β-CD	Randomly methylated β-CD
*S*. *aureus*	*Staphylococcus aureus*
SAED	Selected area electron diffraction
SFS	Sulfisoxazole
α-TC	α-tocopherol
TGA	Thermogravimetric analyses
TR	Triclosan
WAXD	Wide angle-X-ray diffraction
WCA	Water contact angle
XPS	X-ray photoelectron spectroscopy
